# Guiding Framework for Driver Assessment Using Driving Simulators

**DOI:** 10.3389/fpsyg.2017.01428

**Published:** 2017-08-22

**Authors:** Jennifer L. Campos, Michel Bédard, Sherrilene Classen, Jude J. Delparte, Deborah A. Hebert, Nellemarie Hyde, Geoff Law, Gary Naglie, Stephanie Yung

**Affiliations:** ^1^iDAPT Research, Toronto Rehabilitation Institute, University Health Network Toronto, ON, Canada; ^2^Department of Psychology, University of Toronto Toronto, ON, Canada; ^3^Centre for Research on Safe Driving, Lakehead University Thunder Bay, ON, Canada; ^4^Department of Occupational Therapy, University of Florida Gainesville, FL, United States; ^5^Brain and Spinal Cord Rehabilitation Program, Toronto Rehabilitation Institute, University Health Network Toronto, ON, Canada; ^6^Department of Occupational Science and Occupational Therapy, University of Toronto Toronto ON, Canada; ^7^Saint Elizabeth Health Care Toronto, ON, Canada; ^8^Baycrest Health Sciences Toronto, ON, Canada; ^9^Department of Medicine and Institute of Health Policy, Management and Evaluation, University of Toronto Toronto, ON, Canada

**Keywords:** driving simulator, scenarios, outcomes, assessment, evaluation, driving skills, risk assessment, psychomotor performance

Driving simulators are powerful tools for use in research and applications concerned with the evaluation and improvement of driving performance. The value of this technology is contingent upon carefully considering the technical features of the simulator itself (e.g., type of visual display, vehicle control model), the development of appropriate hypothesis-motivated driving scenarios, and the selection of meaningful outcome measures with respect to the questions being addressed and the populations of interest. The Toronto Rehabilitation Institute's iDAPT Centre for Rehabilitation Research recently developed DriverLab, a 7 degrees-of-freedom, motion-based simulator, containing a passenger vehicle, 360 degree visual projection screen, and unique rain and glare simulators (www.idapt.com). To maximize the efficiency and effectiveness of the research conducted within this unique facility, a workshop was organized during which experts across several fields (academia, clinical, industry, government) met in four separate groups to discuss four targeted themes including: (1) use of simulators for driving assessment; (2) effects of drugs on driving safety; (3) effects of automated vehicle technologies (AVTs; e.g., adaptive cruise control) on driving safety; and (4) techniques for mitigating simulator sickness. This paper describes the consensus achieved by the driving assessment group.

The driving assessment group was specifically tasked with characterizing the role of driving simulators in assessing driving performance across a range of applications and populations including individuals with sensory, motor, or cognitive impairments, psychiatric disorders and neurological disorders. The group includes experts in driving evaluations of medically-at-risk drivers (Hyde); clinical assessment of fitness-to-drive (Hebert, Naglie, Law, Classen); and researchers studying the perceptual, cognitive, and emotional factors associated with driving performance and/or simulation design (Bédard, Classen, Campos, Hebert, Yung). The team also includes representation by government organizations that employ a large workforce of occupational drivers (Canada Post) and those involved in road safety initiatives.

## Role for simulators in augmenting traditional office-based and on-road assessments

Driving simulators hold key advantages for identifying risks to driving safety among different driver populations and across driving conditions when compared to office-based tools and on-road testing (Allen et al., 2011; Classen and Brooks, 2014). Common office-based tests of sensory and cognitive function thought to affect driving safety include, for instance: the Useful-Field-of-View test that assesses visual attention (Ball and Owsley, 1993), the Rapid Pace Walk test that measures general physical functioning (Marottoli et al., 1994), and the Montreal Cognitive Assessment that screens for mild cognitive impairment (Nasreddine et al., 2005). Note that the appropriateness of these tools varies as a function of the driver population and outcomes of interest. See, Vrkljan et al. (2011), Classen et al. (2012), and Bennet et al. (2016) for comprehensive reviews and evaluations of these tools. Although performance on these types of office-based tests have been shown to correlate with aspects of on-road and simulated driving performance, simulators offer greater face validity by recreating the multisensory, multidimensional challenges associated with this complex task in more realistic ways.

Compared to on-road testing, simulators are safer, more easily controlled and standardized, and allow for reproducible and easily modifiable conditions and scenarios. Simulators can also provide objective methods of capturing driver-response data, be used with high-risk populations, introduce more challenging environmental conditions, and create more demanding task-based conditions such as multi-tasking.

While even state-of-the-art simulator technologies are currently insufficient to fully determine fitness-to-drive for the purpose of licensing decisions, they have remarkable potential to address a broad range of research questions and to facilitate the screening of at-risk drivers in practice. For instance, driving simulator assessments may serve as a valuable intermediate stage between office-based measures and on-road assessments. By implementing a trichotomization approach of “pass,” “fail,” “indeterminate,” at the simulator stage, this may help to more precisely categorize a driver's risk status (Molnar et al., 2009; Gibbons et al., 2017). When drivers are appropriately categorized during the simulator stage of assessment, the reliance on resource-heavy on-road assessments will be reduced. For a trichotomization approach to be successful with driving simulators, sensitivity and specificity criteria related to pass or fail must be determined. At each stage of this process (office-based → simulator → on-road), customized clinical interventions, training protocols, or driving cessation plans can be initiated as appropriate.

Simulators can also serve as a tool for identifying specific driving skills that could be targeted for intervention and as a means of administering such interventions. Simulators can also be employed to compare performance metrics across different driver populations and to detect changes in the performance of the same individual over time. Repeated driving simulator assessments might be particularly important for monitoring individuals with declining health conditions such as dementia or macular degeneration, individuals with variable symptoms such as bipolar disorder, and individuals taking medications of different types or dosages.

## Necessary requirements in the design and implementation of driving simulator protocols when used for assessment

With driving simulators becoming increasingly accessible due to wider availability, decreased costs, and increasingly specialized expertise, a growing appeal exists to use them widely in both clinical and research contexts. Irrespective of research domains or application, the following important conditions should be considered when using simulators for assessment.

### Maintain a consistent language

Maintaining consistency among terminologies and taxonomies used in protocols, reports, and publications is of great importance. A recent publication in the Transportation Research Board Circular (2016) provides a helpful “*Taxonomy and Terms for Stakeholders in Senior Mobility.”* A lack of uniformity leads to misinterpretation of research findings, inconsistencies in research design implementation, and ineffective translation into practice. For example, authors may indicate that they are assessing “driving safety” when they are actually assessing “driving skills” (i.e., proper vehicle control choices and knowledge of the rules of the road), or “driving abilities” (i.e., sensory, cognitive, motor, functions used during vehicle control).

### Establish fidelity, validity, reliability

To ensure that the simulated driving experience is as comparable as possible to real-world driving experiences, establishing high *fidelity*, including: physical (e.g., motion capabilities), sensory (e.g., image resolution, field of view), and emotional (e.g., fear of adverse events) fidelity is important (Roza, 2005). For driving simulator outcomes to be reflective of what would occur during on-road driving, *validity* must also be established (ecological, content, construct, criterion; Kaptein et al., 1996; Lee et al., 2003; Bédard et al., 2010; Shechtman, 2010). While absolute validity may be difficult to obtain, relative validity is sufficient for many applications (Mullen et al., 2011). Moreover, simulator assessments must demonstrate reproducible results, and therefore *reliability*, including inter-rater and test-retest reliability must be evidenced (Bédard et al., 2010). Classen and Akinwuntan (2017) provide explicit operational definitions of these criteria and how they apply to driving simulation for use in assessment. While there is some limited evidence indicating that driving simulation measurements have moderate-high reliability, less is known about validity (Contardi et al., 2004; Lew et al., 2005; Bédard et al., 2010; Classen and Akinwuntan, 2017). Therefore, more evidence-based research to establish these criteria is warranted. With the advent of more sophisticated driving simulators, this is becoming increasingly possible to achieve.

### Develop hypothesis-driven assessment protocols

Before developing a driving simulation assessment protocol, core objectives must be precisely defined using established conceptual frameworks, theories, and empirical evidence. The design of each study should be hypothesis-driven and the *scenarios* and the *outcome measures* chosen must be rigorously selected and inherently meaningful (see below). Investigators may be inclined to create scenarios that broadly sample from real-world driving experiences to be inclusive; however this approach lacks a clear motivation with respect to the hypotheses being evaluated. Similarly, it is tempting to analyze and report every outcome metric that the simulator software is able to extract. However, the inclusion and interpretation of specific outcome measures should be conceptualized *a priori* rather than discovered *post-hoc*. Exploratory analyses can have great utility in new and developing fields to reveal unanticipated effects, or model multivariate associations that may otherwise be difficult to conceptualize. However, caution should be taken in using results from these approaches to make recommendations regarding driving safety or to establish best practices prior to evaluating them in a prospective, hypothesis-driven manner.

## Scenarios should be customized to the driving behaviors and populations of interest

Driving scenarios have been categorized as targeting “operational level” (e.g., braking, steering), “tactical level” (e.g., maneuvering around obstacles, merging into traffic), and “strategic level” (e.g., mapping out goals and routes) performance based on Michon's hierarchical model of driving behaviors (Michon, 1985; Lindstrom-Forneri et al., 2010; Transportation Research Board Circular, 2016). The design of every simulated drive should deliberately target specific scenario levels based on predictions regarding the tasks for which a given population of drivers would be expected to be most at risk. For example, different tasks would challenge the risk associated with a motor impairment such as using a prosthetic device, vs. a cognitive impairment such as dementia. Driving assessments of those with motor impairments should include scenarios related to operational and tactical level performance given that mechanical or physical behaviors are most likely to be affected. In contrast, driving assessments of those with cognitive impairments might also include scenarios related to strategic level performance given that cognitive skills such as executive functioning and memory are likely to affect these types of driving behaviors.

In order to assess drivers under more challenging situations, layers of increased sensory, motor, or cognitive demands can be introduced. For example, consider the differences in sensory and cognitive load when an older driver negotiates daytime driving (optimal, low load), vs. nighttime driving in the rain (adverse, high load). Importantly, driving researchers should provide detailed descriptions of and justifications for the scenarios used in order to allow for conceptual clarity, reproducibility, and comparisons across studies.

## Outcome measures should be hypothesis-driven

Outcome measures take various forms that uniquely address specific objectives (see Caird and Horrey, 2011 for a summary of common outcome measures). For instance simulator software typically collects kinematic data such as measures of longitudinal/lateral accelerations and turning rate, as well as summary statistics such as number of collisions. Notably, kinematic data provides more detailed information about the spatial and temporal dynamics of behavior compared to summary statistics.

Other outcome measures include assessments performed by trained evaluators such as licensing evaluators, occupational therapists, or driver rehabilitation specialists. These evaluators may observe drivers' behaviors during the simulated driving session or through video recordings. These might include, for instance, standardized demerit-point inventories used by licensing authorities, or other techniques used by driver rehabilitation specialists, such as structured observations of driving behaviors.

Other adjunctive measures that can be used to inform the interpretation of driving simulator performance measures include questionnaires (e.g., driving history and habits; Owsley et al., 1999), subjective rating scales (e.g., driving confidence, anxiety), and physiological response measures (e.g., heart rate, eye movements, brain activity; Johnson et al., 2011; Schweizer et al., 2013). It is also critical to monitor, measure and report common symptoms of simulator sickness (e.g., dizziness, nausea; see the Simulator Sickness Questionnaire Kennedy et al., 1993; Classen et al., 2011). Ultimately, one can evaluate how these adjunctive measures are associated with simulated driving related outcomes. For instance, one must determine whether measures of simulator sickness are associated with measures of driving performance to ensure that the interpretations of the real outcomes of interest are not confounded (Mullen et al., 2010).

## Existing gaps and priorities for future research

In order to fully exploit the exciting potential of driving simulators in driving assessment there is a need to:

- Employ common and consistent operational definitions.
- Establish the validity and reliability of simulator-based assessments.
- Understand how the technological features of the simulator (e.g., field of view, motion capabilities) are associated with fidelity, validity, and reliability measures.
- Use a structured, targeted, hypothesis-driven approach when designing driving assessment scenarios and when interpreting outcome measures.
- Be transparent in reporting null results and negative outcomes (e.g., rates of simulator sickness).

While our team plans to tackle many of these issues using DriverLab by exploiting its unique, high fidelity features, these priorities apply broadly to any research or clinically-based driving simulator that is used for assessment.

## Conclusions

Simulators are emerging as valuable tools for both research and driver assessment applications. The most significant current gaps that have been identified include the need to more firmly establishing criterion validity, ecological validity, and absolute validity across different configurations, applications and populations. The most significant opportunities for utilizing driving simulators occur in the context of assessing at-risk populations, modeling the effects of challenging environmental conditions in more realistic ways (night driving, rain), and evaluating the influence of emerging automated vehicle technologies on driving performance.

## Author contributions

All authors listed have made a substantial, direct and intellectual contribution to the work, and approved it for publication.

### Conflict of interest statement

The authors declare that the research was conducted in the absence of any commercial or financial relationships that could be construed as a potential conflict of interest. The reviewer LB and handling Editor declared their shared affiliation, and the handling Editor states that the process nevertheless met the standards of a fair and objective review.
